# Cell cycle and apoptosis: a map for the GS-NS0 cell line at the genetic level and the link to environmental stress

**DOI:** 10.1186/1753-6561-7-S6-P54

**Published:** 2013-12-04

**Authors:** Chonlatep Usaku, David Garcia Munzer, Efstratios N Pistikopoulos, Athanasios Mantalaris

**Affiliations:** 1 Biological Systems Engineering Laboratory, Centre for Process Systems Engineering, Department of Chemical Engineering, Imperial College London, London, SW7 2AZ, UK

## Background

Large scale mammalian cell culture systems, especially fed-batch systems, are currently utilised to manufacture monoclonal antibodies (MAbs) in order to meet the continuously growing global demand [1]. Nutrient deprivation and toxic metabolite accumulation commonly encountered in such systems influence the cell cycle and trigger apoptosis, resulting in shorter culture times and a lower final MAb titre. Control of the cell cycle has been previously studied in order to achieve higher titre through apoptosis inhibition by bcl-2 overexpression and cell cycle arrest in G_1_/G_0 _by p21 transfection. However, the above mentioned strategies have not always been successful; no improvement in titre was often observed though bcl-2 over-expression helped prolong the culture viability whereby the majority of cells were arrested at G_1_/G_0 _to avoid apoptosis [2-4]. Thus, a systematic insight of the dynamic relation between metabolic stress, cell cycle and apoptosis is still required. To this end, we aim to establish a novel map of the dynamic interplay between cell cycle and apoptosis at the genetic level, and provide a link with the culture conditions at the metabolic level.

## Materials and methods

Batch culture of GS-NS0 producing a cB72.3 MAb was performed. Cell density and viability was quantified using the dye exclusion method. Extracellular glucose, glutamate, lactate and ammonium were quantified using Bioprofile 400 (Nova Biomedical, Waltham, USA). The extracellular antibody was measured using ELISA. DNA staining and Annexin V/PI assay was used to quantify the fraction of cells in each cell cycle phase as well as the degree of apoptosis. The measurement of both apoptosis and cell cycle related gene expression was conducted using real-time PCR.

## Results and discussion

Our results showed a clear link between the environmental factors and gene expression. The batch cultures started with a high fraction of cells in the G_1_/G_0 _phase, which rapidly left this state in order to join the proliferating population. Soon after, glutamate deprivation occurred at around 50 h of culture, whereby *atf5 *upregulation peaked (50% higher) suggesting that glutamate deprivation is among the first factors that introduce metabolic stress, in agreement with previous results [5]. The upregulation of *atf5 *triggered the upregulation of *bcl-2 *(which followed at around 90 h). After the batch cultures reached their maximum cell density (which occurred roughly the same time as the glutamate exhaustion), the onset of an increasing early apoptotic cell population was observed - around 10%. Together with the high cell density, *casp8 *was upregulated (100% increase). Consequently, the expression of *casp3 *followed a similar trend with a lag of few hours as its protein, caspase-3, is one of downstream targets of caspase-8 and a final executor of the apoptosis pathways [6]. In addition, *trp53bp2 *showed a similar trend to *casp3*. These results suggest that apoptosis could initially occur via the death receptor pathway as marked by the *casp8 *upregulation, which might be induced by the glutamate exhaustion and/or the cell density peak. However, given that the *trp53bp2 *upregulation happened later than that of *casp8 *suggests that apoptosis in the later stages of culture might also occur through the mitochondrial pathway and it could also be triggered by other lethal signals (e.g. high level of lactate accumulation). As soon as the onset of apoptosis occurred, the upregulation of *p21 *was also observed (300% increase) and this happened simultaneously with the *bcl-2 *upregulation. Since it was reported that Bcl-2 protein helps facilitate cell cycle arrest at G_1_/G_0 _phase and an increase in G_1_/G_0 _cell fraction was observed later in the death phase of culture, this could suggest that the *bcl-2 *upregulation may underlie the *p21 *upregulation and the cell cycle arrest at G_1_/G_0 _phase and this could be a mechanism to avoid apoptosis [7].

## Conclusions

These findings set a map of the cell cycle and apoptotic timing and magnitudes of the events from the genetic level and their links to the environmental conditions, which can be used to gain insight of the GS-NS0 cultures. By looking at the map, we can systematically analyse cellular responses to the environmental conditions which may have detrimental effect on the culture and utilise the result of the analysis to tackle the culture issues way before the final executors, but at the genetic level. Ultimately, the goal is to utilize mathematical models that will help to establish new strategies in order to achieve a longer cultivation period, high viability and increased MAb titre.
